# Evaluation of Electrospun PCL-PIBMD Meshes Modified with Plasmid Complexes *in Vitro* and *in Vivo*

**DOI:** 10.3390/polym8030058

**Published:** 2016-02-23

**Authors:** Yakai Feng, Wen Liu, Xiangkui Ren, Wei Lu, Mengyang Guo, Marc Behl, Andreas Lendlein, Wencheng Zhang

**Affiliations:** 1 School of Chemical Engineering and Technology, Collaborative Innovation Center of Chemical Science and Chemical Engineering (Tianjin), Tianjin University, Tianjin 300072, China; yakaifeng@tju.edu.cn (Y.F.); 18202520278@163.com (W.L.); care1990@tju.edu.cn (W.L.); 15002257297@163.com (M.G.); 2 Tianjin University–Helmholtz-Zentrum Geesthacht, Joint Laboratory for Biomaterials and Regenerative Medicine, Tianjin 300072, China; 3 Key Laboratory of Systems Bioengineering of Ministry of Education, Tianjin University, Tianjin 300072, China; 4 Institute of Biomaterial Science, Berlin Brandenburg Center for Regenerative Therapies, Helmholtz-Zentrum Geesthacht, Kantstr. 55, 14513 Teltow, Germany; marc.behl@hzg.de; 5 Tianjin University–Helmholtz-Zentrum Geesthacht, Joint Laboratory for Biomaterials and Regenerative Medicine, Kantstr. 55, 14513 Teltow, Germany; 6 Department of Physiology and Pathophysiology, Logistics University of Chinese People’s Armed Police Force, Tianjin 300162, China

**Keywords:** electrospun meshes, microparticles, endothelialization

## Abstract

Functional artificial vascular meshes from biodegradable polymers have been widely explored for certain tissue engineered meshes. Still, the foreign body reaction and limitation in endothelialization are challenges for such devices. Here, degradable meshes from phase-segregated multiblock copolymers consisting of poly(ε-caprolactone) (PCL) and polydepsipeptide segments are successfully prepared by electrospinning and electrospraying techniques. The pEGFP-ZNF580 plasmid microparticles (MPs-pZNF580) were loaded into the electrospun meshes to enhance endothelialization. These functional meshes were evaluated *in vitro* and *in vivo*. The adhesion and proliferation of endothelial cells on the meshes were enhanced in loaded mesh groups. Moreover, the hemocompatibility and the tissue response of the meshes were further tested. The complete tests showed that the vascular meshes modified with MPs-pZNF580 possessed satisfactory performance with an average fiber diameter of 550 ± 160 nm, tensile strength of 27 ± 3 MPa, Young’s modulus of 1. 9 ± 0.2 MPa, water contact angle of 95° ± 2°, relative cell number of 122% ± 1% after 7 days of culture, and low blood platelet adhesion as well as weak inflammatory reactions compared to control groups.

## 1. Introduction

The morbidity and mortality caused by cardiovascular disease are expected to increase as the world develops into an old-age society [1]. Diseases of peripheral and coronary arteries are major causes for death [2]. Cardiovascular diseases such as atherosclerosis may result in obstruction of blood vessels and tissue ischemia, thus leading to a massive demand for blood vessel substitutes every year [3]. The available options for such substitutes include autologous grafts, allografts, xenografts, artificial prostheses or synthetic vascular grafts made from expanded polytetrafluoroethylene and polyethylene terephthalate [4,5,6]. However, the use of autografts and allografts is limited due to the lack of tissue donors, the risk of disease transmission and anatomical variability [7]. As for xenografts, they usually suffer from their relatively short life span and immunogenicity [8,9].

Compared to autologous grafts, allografts and xenografts, the application of artificial blood vessels is increasing significantly [10,11]. The performance of artificial blood vessels is satisfactory in large-diameter vessels (≥6 mm), but many problems such as thrombosis, intimal hyperplasia, and low long-term patency rate are encountered in small-diameter vascular grafts (<6 mm) [12,13,14]. Tissue engineering possesses immense potential for the fabrication of artificial small-diameter blood vessels, as progress has been made in constructing various components of the cardiovascular system, including heart valves and cardiac muscle [15]. In order to promote the biocompatibility of artificial small-diameter vascular grafts and improve their *in vivo* usage, surface modifications with various types of capturing ligands, including peptides, antibodies, magnetic molecules, oligosaccharides and aptamers, have been explored by researchers [16,17,18,19]. Among these modifications, the aim of rapid endothelialization is of great importance, as many implant failures of artificial blood vessels are closely linked with the poor endothelialization [20,21]. Del Gaudio *et al.* [22] reported that the adsorption of vascular endothelial growth factor (VEGF) on genipin cross-linked gelatin mats boosted and induced early angiogenesis. Electrospun poly(ε-caprolactone) (PCL) functionalized by arginine-glycine-aspartic acid (RGD) exhibited improved hydrophilicity, enhanced adhesion and spreading of endothelial cells (ECs) [23,24]. Encapsulated nanoparticles containing vascular endothelial growth factor plasmids (pVEGF) and basic fibroblast growth factors plasmids (pbFGF) in electrospun meshes led to significantly higher density of mature blood vessels [25]. Besides the above genes, our group has employed ZNF580 gene complexed with biodegradable microparticles (MPs) to promote the proliferation of ECs [26,27,28].

Physicochemical properties of biodegradable materials also play a crucial role in the performance of artificial blood vessels. Various factors should be considered for material choice, including biomechanical properties, morphology, biodegradation rate and immunogenicity. It is known that polyglycolic acid (PGA), polylactic acid (PLA), PCL and their copolymers are the most extensively investigated polymeric materials for biodegradable meshes in vascular tissue engineering applications [29,30].

A common feature of these biodegradable polymers is the accumulation of acidic degradation products during degradation process, which can cause inflammatory and foreign body reactions *in vivo* following the acidification of surrounding tissues [31]. The reconstruction of cardiovascular tissue engineering relate to the combination between grafts degradation during *in vivo* conditioning and the cell productivity *in situ*, so that the construct integrity is obtained from the newly formed tissue [32]. Hence, it is of great importance to decrease the side effects caused by grafts degradation. Synthetic multiblock copolymers composed of PCL and poly(isobutyl-morpholine-2,5-dione) (PIBMD) segments have been identified as an attractive candidate material for mesh materials because the *α*-amino acids formed along degradation may buffer the hydroxy acids, therefore minimizing inflammation reactions during degradation of implanted meshes [33,34].

Recently, we have developed a polycationic gene carrier functionalized with targeting REDV peptide for carrying the pEGFP-ZNF580 plasmid. These targeted complexes exhibited good cyto-compatibility and enhanced the transfection and migration capability of ECs significantly [35]. It is of great interest to know whether it is possible to introduce the targeted complexes onto the surface of artificial vascular meshes via electrospinning and electrospraying techniques to improve the proliferation and migration of ECs.

Here, electrospraying was applied to recruit MPs-pZNF580 onto the surface of electrospun PCL-PIBMD meshes for enhancing endothelialization. The morphological characterization was examined by scanning electron microscopy (SEM), and the tensile mechanical behavior of PCL-PIBMD fibrous meshes was investigated by tensile tests. The behavior of ECs including cell adhesion and relative cell number was evaluated *in vitro*. Because the implanted mesh materials would have direct contact with blood, the hemocompatibility of electrospun PCL-PIBMD meshes was also assessed via platelet tests. The tissue response of electrospun PCL-PIBMD meshes was clarified after subcutaneous implantation into Sprague-Dawley rats via hematoxylin and eosin (H&E) staining.

## 2. Experimental Section

### 2.1. Materials

PCL-PIBMD was synthesized from PCL-diol and PIBMD-diol with 2,2,4-, and 2,4,4-trimethylhexamethylene diisocyanate (TMDI) as a coupling agent in previous work [36]. Tetrahydrofuran (THF), chloroform (CHCl_3_), dimethyl sulfoxide (DMSO) and phosphate buffered saline (PBS) were purchased from Tianjin Jiangtian Chemical Technology Co., Ltd., Tianjin, China. Stannous octoate (Sn(Oct)_2_), ε-caprolactone (99%, CL), branched PEI (*M*_w_ = 1800 Da), methoxy poly (ethylene glycol) ether (mPEG, *M*_w_ = 2000 Da) and fluorescein diacetate (FDA) were all purchased from Sigma-Aldrich (St. Louis, MO, USA). l-Lactide (LA) and glycolide (GA) was obtained from Foryou Medical Device Co., Ltd. (Huizhou, China). Orthopyridyl disulfide-PEG-*N*-hydroxysuccinimide (OPSS-PEG-NHS, *M*_n_ = 2294 Da, the *M*_n_ of PEG is 2000 Da, 99%) was purchased from JenKem (Beijing, China) Technology Co., Ltd. CREDVW peptide was supplied by GL Biochem (Shanghai, China) Ltd. H&E was purchased from Sigma-Aldrich (St. Louis, MO, USA). The human endothelial cell hybridoma line, EA.hy926, was obtained from the Cell Bank of Typical Culture Collection of Chinese Academy of Sciences (Shanghai, China). The pZNF580 plasmid was preserved by the Department of Physiology and Pathophysiology, Logistics University of Chinese People’s Armed Police Force. Sprague-Dawley rats (male, 150–200 g) were purchased from the Laboratory Animal Center of the Academy of Military Medical Sciences (Beijing, China).

### 2.2. Preparation of MPs and MPs-pZNF580 Complexes Suspension

The MPs suspension was prepared as our previous work described [35,37,38]. Briefly, the diblock copolymer of mPEG-*b*-P(LA-*co*-CL) was prepared by ring-opening polymerization (ROP) of mPEG, LA and GA monomers with Sn(Oct)_2_ as catalyst. Secondly, copolymer of mPEG-*b*-P(LA-*co*-CL)-*g*-PEI was synthesized from the diblock copolymer and PEI. Then, CREDVW peptide was conjugated with mPEG-*b*-P(LA-*co*-CL)-*g*-PEI using the linker of orthopyridyl disulfide-PEG-*N*-hydroxysuccinimide (OPSS-PEG-NHS). The MPs composed of mPEG-*b*-P(LA-*co*-CL)-*g*-PEI-REDV were created via nano-precipitation method. MPs-pZNF580 complexes were prepared by adding pEGFP-ZNF580 plasmid solution to the obtained MPs suspension at N/P molar ratio of 10, which was suitable for cellular uptake [36]. The average hydrodynamic diameter of MPs used here was between 30–300 nm and zeta potential was about 20 mV. The hydrodynamic diameter of the MPs was analyzed by dynamic light scattering (DLS) and the values of zeta potential were measured using a Zetasizer 3000HS (Malvern Instrument, Inc., Worcestershire, UK) at the wavelength of 677 nm with a constant angle of 90°.

### 2.3. Electrospinning Process

PCL-PIBMD was dissolved in CHCl_3_/THF (3:1, *v*/*v*) to prepare 10% (*w*/*v*) mixed solution. Then, the mixture was stirred for 6 h to form a homogeneous solution at room temperature. The prepared PCL-PIBMD spinning solution was filled with 2.0 mL glass syringes fitted with 0.7 mm diameter needles to fabricate the fibers. The applied voltage, feed rate, and target distance were 20 kV, 0.5 mL/h, and 15 cm, respectively. The electrospun fibers were collected on a metal plate covered with aluminum foil. Afterwards, the fibrous meshes were then dried in a vacuum oven at room temperature for 24 h before use.

In order to investigate the effect of MPs-pZNF580 on the ECs *in vitro*, the same concentration and volume (0.4 mg/mL, 0.8 mL) of MPs and MPs-pZNF580 were used [39]. Briefly, the PCL-PIBMD fibrous meshes modified with MPs or MPs-pZNF580 were prepared by electrospraying MPs suspension or MPs-pZNF580 complexes suspension onto the surface of electrospun meshes, in which MPs or MPs-pZNF580 were simultaneously collected onto the aluminum foil coated with PCL-PIBMD fibers. In the preparation process, the MPs suspension or MPs-pZNF580 complexes suspension was electrosprayed under the same conditions with applied voltage of 18 kV, feed rate of 0.5 mL/h, and target distance of 15 cm.

### 2.4. Characterization of Electrospun Fibrous Meshes

#### 2.4.1. Analysis of the Surface Morphology of Electrospun Fibrous Meshes

SEM (Hitachi S-4800, Tokyo, Japan) was utilized to show the surface morphology of meshes at an accelerating voltage of 10 kV. The meshes were coated with gold using a sputter coater for 90 s (Hitachi, E-1045, Tokyo, Japan). The fiber diameter of the electrospun fibers was measured by Digimizer software from the SEM micrographs in original magnification of 10,000×. At least 100 fibers in different SEM images were selected for the analysis. The porosity of each mesh was calculated from corresponding SEM images through Photoshop software.

#### 2.4.2. FT-IR Measurements

Attenuated total reflection Fourier Transform Infrared spectroscopic analysis (ATR-FTIR, Bio-Rad FTS-6000, Hercules, CA, USA) of electrospun meshes was performed to characterize the composition of the fibrous meshes with a spectral range of 4000–600 cm^−1^. Each spectrum was acquired in reflectance mode on a diamond crystal by the accumulation of 32 scans with a resolution of 2 cm^−1^.

#### 2.4.3. Mechanical Properties Tests

The mechanical properties of electrospun meshes, which were cut into rectangles with a length of 20 mm, a width of 5 mm and a thickness in the range between 350 μm and 470 μm determined with thickness meter, were measured by means of a tensile tester (WDW-02, Changchun, China). The meshes were immersed in PBS for 4 h and then fixed on the clamp of the testing system. Maximum stress and strain at rupture were measured. The Young’s modulus representing the elasticity was obtained by determining the slope of the stress–strain curve in the elastic region. Five samples of each electrospun meshes were tested to calculate an average value.

#### 2.4.4. Static Water Contact Angle

The fibrous meshes were cut into rectangular shapes (20 mm × 10 mm) for the static water contact angle (WCA) measurement using a contact angle instrument (Krüss Easy Drop goniometer, Hamburg, Germany) by sessile drop method at room temperature. The droplet size was set at 3 μL and the instantaneous WCA was recorded within 0.5 s. Five points of each sample were tested to calculate average values of static water contact angle.

### 2.5. Biological Evaluation

#### 2.5.1. Culture of EA.hy926 Cells

EA.hy926 cells are a hybrid cell line of human endothelium and lung carcinoma cells [40], which not only conserve the characteristics of endothelial cells, but also can be subcultured. To investigate the function of electrospun PCL-PIBMD meshes modified with plasmid complexes towards endothelial cells, we used EA.hy926 cells. Prior to cell seeding, fibrous meshes were soaked in 75% ethanol for 1 h, and then washed 3 times with PBS. A 20-μL EA.hy926 cell suspension (5 × 10^5^ cells/mL) was seeded onto the pretreated discs. Then, 200 μL culture medium DMEM (Gibco, Waltham, MA, USA), supplemented with 10% fetal bovine serum and 1% penicillin and streptomycin, was added to the EA.hy926 cells suspension. The culture conditions of EA.hy926 cells were kept at 37 °C under 5% CO_2_.

#### 2.5.2. Cell Proliferation Assay

EA.hy926 cells were seeded at a density of 1 × 10^4^ cells/well onto the pretreated meshes in 96-well culture plate for 1, 3 and 7 days. At the end of the culture period, each sample was incubated with the thiazolyl blue for 4 h. The purple formazan reaction product was then dissolved by adding 150 μL DMSO and vibrated slightly for 10 min at room temperature. Finally, the optical density of the wells was determined at a test wavelength of 490 nm using a plate reader (Bio-Rad Laboratories).

#### 2.5.3. Fluorescence Assay

The cell-seeded meshes were obtained as above, and then the meshes were incubated in 500 µg/mL FDA solution diluted with PBS for 30 min at 37 °C for staining. Afterwards, meshes were imaged using the inverted fluorescent Microscope (Olympus CKX41, Tokyo, Japan). Cell counting data were acquired from counting total number of cells in three separate fiber pieces at three different time points (1, 3, and 7 days). The total number of cells was presented as number of cells per day of culture.

#### 2.5.4. Platelet Adhesion Assay

The platelet adhesion test was performed according to the method of Chung and Rhee [41]. Briefly, a sample of 20 μL of platelet-rich plasma (PRP, 10^8^ platelets/mL) was carefully dropped on the fibrous meshes placed in 24-well polystyrene plates. After incubation for 2 h at room temperature, the meshes were carefully rinsed several times in PBS (pH = 7.4) to remove non-adhering platelets. Adherent platelets on the meshes were preserved with 2.5% glutaraldehyde/PBS solution for 30 min, followed by dehydration procedure using a series of ethanol-water mixtures (30%, 50%, 70%, 90% and 100%) for 30 min, respectively. The adhered platelets on the meshes were observed by a scanning electron microscope (SEM, Hitachi S-4800, Tokyo, Japan).

#### 2.5.5. Subcutaneous Implantation

All animal procedures were approved by the Animal Care Committee and in accordance with the regulations for the administration of affairs concerning experimental animals in Logistics University of Chinese People’s Armed Police Force. Prior to the implantation of meshes, they were treated with a 70% ethanol solution overnight and then left in sterile filtered PBS containing 4% penicillin/streptomycin. Eighteen Sprague-Dawley rats (male, 150–200 g) were used for this study. They were randomly assigned to three groups (six in each group). In PCL-PIBMD group, two disc specimens (Φ = 6 mm) were placed in the dorsal subcutaneous pocket symmetrically per rat. PCL-PIBMD/MPs and PCL-PIBMD/MPs-pZNF580 were implanted in the same way. At the end of the subcutaneous implantation period (7 days), the implanted meshes (6 samples per time point) with surrounding tissue were harvested and fixed in 10% buffered formalin, solution for 2 days. Once the dehydration procedure was completed in ascending series of ethanol, each specimen was embedded in paraffin and cut into sections with thicknesses of 5 μm. Then the sections were deparaffinized, rehydrated and stained with H&E to observe the infiltration of inflammatory cells under an inverted microscope (Nikon Eclipse TE2000-U, Kanagawa, Japan).

### 2.6. Statistical Analysis

All quantitative results were obtained on the average of measurements from three samples. Data were expressed as the mean ± standard deviation (SD). Statistical analysis was performed using one-way ANOVA, performed with a computer statistical program (PASW Statistics 18). Fisher LSD (least significance difference) test was performed to determine statistical significance between groups. A value of *p* < 0.05 was considered to be statistically significant.

## 3. Results and Discussion

### 3.1. Morphology and Characterizations of Fibrous Meshes

**Figure 1:**
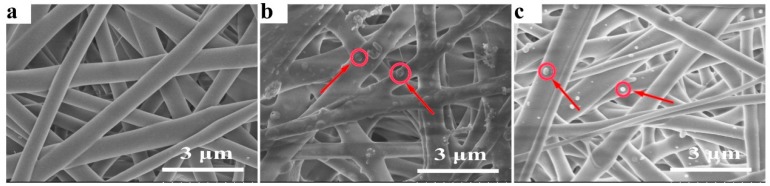
SEM micrographs of electrospun fibrous meshes: (**a**) PCL-PIBMD meshes; (**b**) PCL-PIBMD/MPs meshes; and (**c**) PCL-PIBMD/MPs-pZNF580 meshes. MPs and MPs-pZNF580 were marked by red circles and arrows.

During electrospinning process, the viscoelastic solution is stretched into nano/microfibers via a high electrostatic force [42]. Many parameters, including the concentration of the feed solution, their viscosity or electrical conductivity, the molecular weight of the electrospun polymer or its surface tension, the voltage applied, the flow rate, the distance of the collector from the tip, and even environmental parameters such as humidity and temperature, have an impact on the morphology of electrospun fibers [43,44]. Uniform, continuous and stable fibers were obtained at an applied voltage of 20 kV, a feed rate of 0.5 mL/h, and a target distance of 15 cm. The morphology of various electrospun meshes with approximately 100 μm thickness was characterized by SEM in Figure 1. Figure 1a shows the morphology of the pure PCL-PIBMD fibers. The PCL-PIBMD fibers with a diameter of 560 ± 170 nm were formed with random orientation and without beads. The average fiber diameters of the PCL-PIBMD/MPs and PCL-PIBMD/MPs-pZNF580 were similar: the PCL-PIBMD/MPs fibers had an average diameter of 550 ± 130 nm (Figure 1b), whereas the PCL-PIBMD/MPs-pZNF580 fibers showed an average diameter of 550 ± 160 nm (Figure 1c). Figure 1 clearly demonstrates that the fiber diameters were uneven. This phenomenon may be attributed to micro-phase separation of the block copolymer during electrospinning [45]. Due to the surface tension difference of the components, the diblock polymer solution had a tendency to form two phases, namely, PCL-enriched phase and PIBMD-enriched phase, thus resulting in the non-uniform fiber diameters. From the other perspective, the results also suggested that loading MPs or MPs-pZNF580 did not affect the diameter significantly. In addition, the porosity of PCL-PIBMD, PCL-PIBMD/MPs and PCL-PIBMD/MPs-pZNF580 meshes was 64% ± 14%, 33% ± 8% and 57% ± 6%, respectively. Figure 1b,c confirmed the successful introduction of MPs or MPs-pZNF580 onto the surface of meshes. Furthermore, MPs-pZNF580 appeared to be smaller than MPs, and the declining trend was mainly due to the electrostatic force. The MPs-pZNF580 complexes became compact entities where DNA molecules were highly compressed [46].

### 3.2. ATR-FTIR Analysis

**Figure 2:**
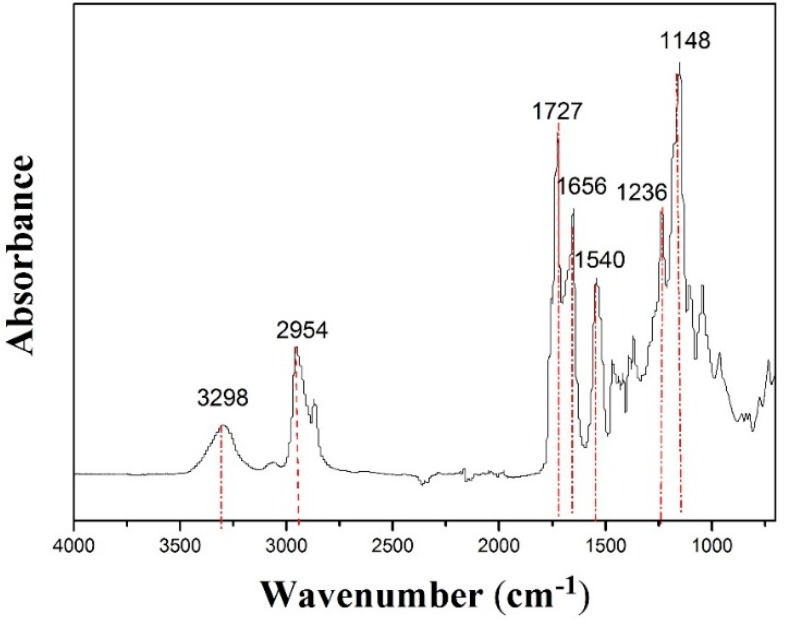
ATR-FTIR of electrospun PCL-PIBMD fibrous meshes.

The FTIR spectra were employed to characterize the functional groups of the PCL-PIBMD fibrous meshes. As shown in Figure 2, at 3298 cm^−1^ were typical bands of –NH– in an IBMD segment and the signal at 2954 cm^−1^ was assigned to –CH_2_– stretching vibration peak. Carbonyl groups (–C=O) are one of the most sensitive markers of polypeptide secondary in the FTIR spectrum [47]. In the FTIR spectrum of PCL-PIBMD fibrous meshes, 1727 and 1656 cm^−1^ were the characteristic peaks of –C=O stretching vibration. Especially, 1727 cm^−1^ referred to the ester bond (–COO–) and 1656 cm^−1^ was resulted from amide bond (–CO–NH–), which was due to p-π conjugation that reduced stretching vibration frequency of –C=O [47]. Moreover, 1236 cm^−1^ was the bending vibration in the structure of –C=O. And the absorption peaks at 1148 and 1540 cm^−1^ were linked with –C–O– stretching vibration and –CH_3_ bending vibration, respectively. These results reflected the structure of PCL-PIBMD directly.

### 3.3. Mechanical Properties of Fibrous Meshes

**Figure 3:**
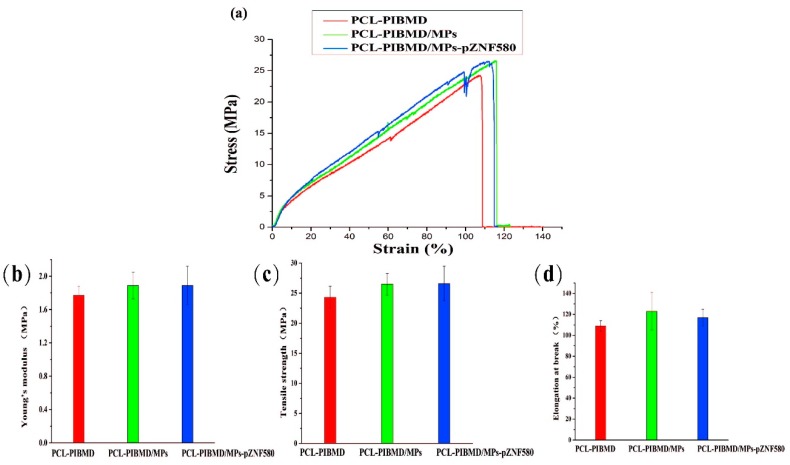
Stress–strain curves for electrospun PCL-PIBMD, PCL-PIBMD/MPs and PCL-PIBMD/MPs-pZNF580 meshes (**a**); Mechanical properties were evaluated: (**b**) Young’s modulus; (**c**) Tensile strength; and (**d**) Elongation at break. Error bars represent mean ± standard deviation (SD) (*p* > 0.05).

Among blood vessels substitutes, where a load bearing structure is required, meshes need to demonstrate sufficient tensile strength in vascular tissue engineering to meet the demands in practical application [48]. Figure 3a shows the stress–strain curves of PCL-PIBMD, PCL-PIBMD/MPs and PCL-PIBMD/MPs-pZNF580 meshes. The correlated data, including Young’s modulus, tensile strength and elongation at break are plotted in Figure 3b–d. The mechanical data showed no significant difference of these meshes before and after electrospraying the microparticles (Fisher LSD test was performed to determine statistical significance between groups, *p* > 0.05). Moreover, PCL-PIBMD meshes were nearly five times stiffer than electrospun PCL meshes [49]. As we know, fresh carotid arteries possess a tensile strength varying from 1.76 to 2.64 MPa and elongation at break varying from 110% to 200% [50]. Thus, the three kinds of vascular meshes prepared here can satisfy the application requirements in mechanical properties.

### 3.4. WCA of Fibrous Meshes

Hydrophilicity needs to be considered for a comprehensive evaluation of biomaterials [51]. Many studies have demonstrated that neither very hydrophobic nor very hydrophilic substrates were favorable to promote cell adhesion and proliferation [52]. WCA measurements were performed to investigate the hydrophilicity of meshes. PCL-PIBMD fibrous meshes exhibited mildly hydrophobic characteristics with a water contact angle value of 109° ± 8°. The surface hydrophilicity was slightly enhanced after introducing MPs with water contact angle values of 95° ± 2°, 95° ± 2° for PCL-PIBMD/MPs and PCL-PIBMD/MPs-pZNF580, respectively (Fisher LSD test was performed to determine statistical significance between groups, * *p* < 0.05, PCL-PIBMD/MPs *vs.* PCL-PIBMD and PCL-PIBMD/MPs-pZNF580 *vs.* PCL-PIBMD). Notably, the water contact angle value of PCL-PIBMD/MPs and PCL-PIBMD/MPs-pZNF580 was almost the same, which was lower than that of PCL-PIBMD meshes. We speculated it resulted from the MPs loaded on the surface of electrospun meshes, which combined with hydrophilic mPEG [53]. Hence, the introduction of MPs to the surface of PCL-PIBMD meshes not only preserved pEGFP-ZNF580 plasmids from being deactivated or denatured, but also provided an approach to improve the hydrophilic features of electrospun PCL-PIBMD meshes.

### 3.5. Morphology of EA.hy926 Cells on PCL-PIBMD/MPs Meshes

**Figure 4:**
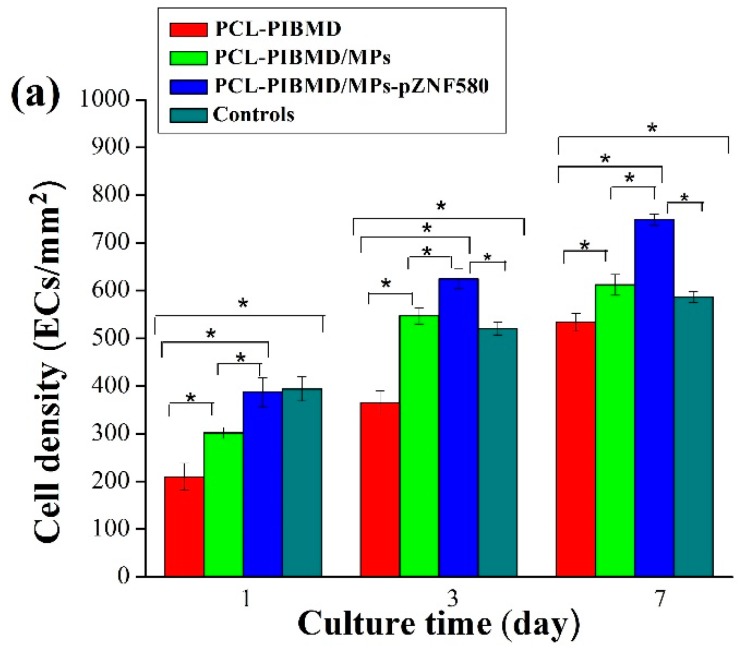

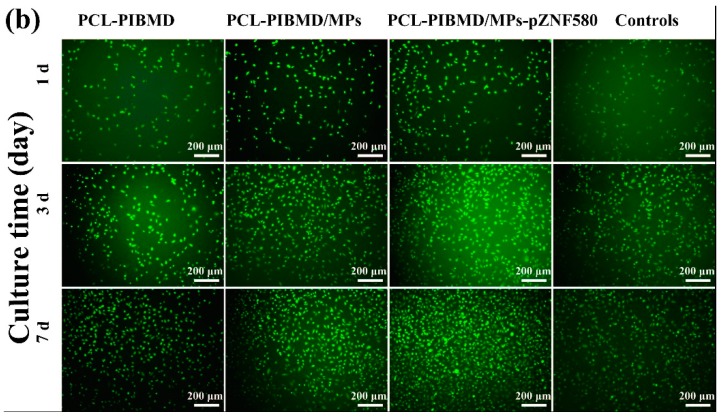
(**a**) EA.hy926 cells density (number of cells/mm^2^). Error bars represent mean ± standard deviation (SD) (Fisher LSD test was performed to determine statistical significance between groups, * *p* < 0.05); (**b**) Fluorescence micrographs of EA.hy926 cells adhesion on PCL-PIBMD, PCL-PIBMD/MPs and PCL-PIBMD/MPs-pZNF580 meshes after 1, 3, and 7 days. Cells without treatment were taken as controls.

**Figure 5:**
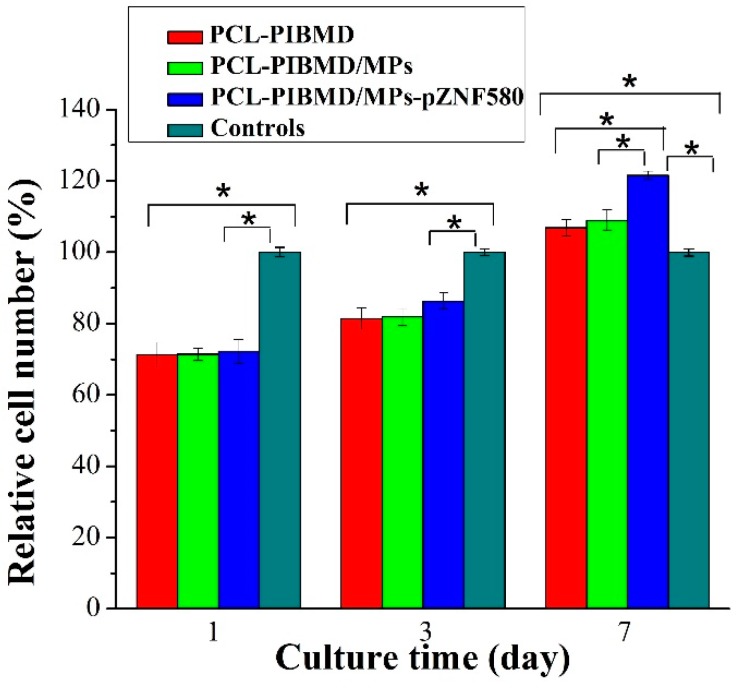
The relative cell number of EA.hy926 cells cultured on PCL-PIBMD, PCL-PIBMD/MPs, and PCL-PIBMD/MPs-pZNF580 meshes for 1, 3, and 7 days. Error bars represent mean ± standard deviation (SD) (Fisher LSD test was performed to determine statistical significance between groups, * *p* < 0.05).

The cell morphology and the contact between the cells and the different meshes after culturing for three different time periods (1, 3, and 7 days) were analyzed by fluorescence assays [54] to understand the effect of the functional MPs loaded on the surface of PCL-PIBMD meshes. Cells without treatment were taken as control group. The EA.hy926 cells adhesion and spread results are shown in Figure 4. The results show that EA.hy926 cells were well adhered and grew to cover a majority of these surfaces. On the first day, the morphology of EA.hy926 cell adhesion was similar on all surfaces. After 3 days of culture, morphology as well as cell numbers of EA.hy926 cells appeared different on individual surfaces. After 7 days of culture, the diversity of EA.hy926 cell growth status among the various meshes became more apparent. Notably, PCL-PIBMD/MPs-pZNF580 meshes were more effective in EA.hy926 cell adhesion and spreading. The cell density (number of cells/mm^2^) plot directly demonstrated that the number of EA.hy926 cells on PCL-PIBMD/MPs-pZNF580 meshes was larger than that of PCL-PIBMD and PCL-PIBMD/MPs (Figure 5). The introduction of MPs could obviously enhance the cell adhesion and spreading, which might be caused by the REDV component present in the MPs structure [55,56,57,58]. Moreover, ECs showed more preference to PCL-PIBMD/MPs-pZNF580 meshes than that of PCL-PIBMD/MPs meshes in the same time intervals. This could result from the expression of pEGFP-ZNF580 plasmids, which was capable of improving the proliferation and migration of ECs [59].

### 3.6. Proliferation of EA.hy926 on PCL-PIBMD/MPs Meshes

The MTT assay enables assessment of the metabolically active cells [60]. In order to be used in tissue engineering applications, the potential vascular meshes should facilitate ECs adhesion, growth and proliferation [61]. The results of MTT assay are shown in Figure 5. Cells without treatment were used as control group. The sample size for cell culture assays is nearly 0.33 cm^2^ matching with 96-well cell culture plate. The relative cell number of EA.hy926 on the PCL-PIBMD meshes after 7 days was 107% ± 2%, which indicated that mesh materials had good biocompatibility. Moreover, the relative cell number of EA.hy926 cells on the PCL-PIBMD/MPs-pZNF580 meshes in the same condition was 122% ± 1%, which was highest among the three vascular meshes. This result was in agreement with that of fluorescence assay, and could be attributed to the cooperative effect of REDV and pEGFP-ZNF580 plasmids.

### 3.7. Platelet Adhesion Examination

**Figure 6:**
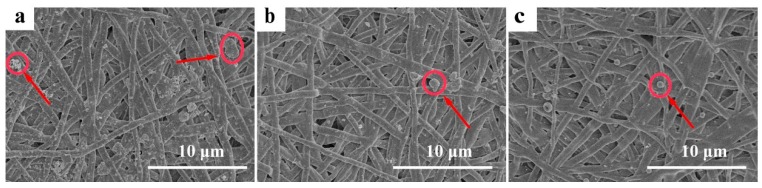
SEM images of platelet adhesion on fibrous meshes: (**a**) PCL-PIBMD meshes; (**b**) PCL-PIBMD/MPs meshes; and (**c**) PCL-PIBMD/MPs-pZNF580 meshes. In the case of PCL-PIBMD, the adhered platelets showed more aggregation, as indicated by red arrows and circles.

To evaluate the hemocompatibility of artificial electrospun meshes, the platelet adhesion tests were taken with the blank PCL-PIBMD meshes as the control. The platelet adhesion on the various meshes was analyzed by SEM (Figure 6). The results showed PCL-PIBMD/MPs and PCL-PIBMD/MPs-pZNF580 surface exhibited relatively lower platelet adhesion than that of PCL-PIBMD after a contact with PRP within 2 h. Although the immobilized plasmids had no direct inhibitory effect on platelet adhesion, the delivery vector of the plasmids composed of hydrophilic mPEG was favorable for hindering platelet adhesion [62]. Moreover, the adhered platelets on PCL-PIBMD/MPs and PCL-PIBMD/MPs-pZNF580 surface behaved round morphology. In contrast, PCL-PIBMD surface adsorbed more platelets and most of them were activated (encircled in red in Figure 6). However, the reason on inhibiting platelet adhesion presented by electrospun PCL-PIBMD/MPs and PCL-PIBMD/MPs-pZNF580 meshes is still unclear. Clearly, the designed PCL-PIBMD/MPs-pZNF580 vascular meshes helped for the rapid endothelialization as well as platelet inhibition so as to meet clinical demand.

### 3.8. Tissue Response of Meshes in Subcutaneous Implantation

**Figure 7:**
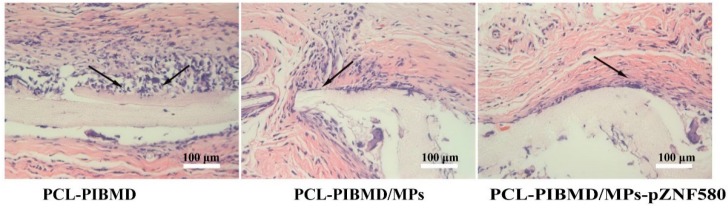
H&E micrographs of subcutaneous implanted PCL-PIBMD, PCL-PIBMD/MPs and PCL-PIBMD/MPs-pZNF580 specimens for 7 days. The inflammatory cells were marked by black arrow.

Tissue compatibility is an essential factor of composite meshes. In order to determine the feasibility of using electrospun vascular meshes for application, tissue compatibility was further evaluated via H&E staining of implanted mesh material for evidence of abnormal tissue response in a subcutaneous implantation mode [63]. Histological analysis of the retrieved implants was performed to evaluate inflammatory cell infiltration during the acute inflammation stage (7 days). The representative micrographs of histological sections of explanted samples are shown in Figure 7.

All the explanted meshes showed the infiltration of inflammatory cells to different degrees. A large number of recruited neutrophil infiltrates and multinucleated giant cells were observed in tissue surrounding the PCL-PIBMD. Nevertheless, when the tissues contact with the meshes modified with MPs-pZNF580, the acute inflammatory reaction after 7 days was slightly diminished.

The previous study suggested that decellularized porcine meshes could generate a strong inflammatory response during the same period [64]. However, *in vivo* assay results further supported the good tissue compatibility of PCL-PIBMD/MPs-pZNF580 meshes. We presumed that the expression of pEGFP-ZNF580 plasmids successfully decreased the infiltration of inflammatory cells compared to the PCL-PIBMD and PCL-PIBMD/MPs meshes via NF-κB signaling pathways [65]. To this end, we anticipated that the introduction of MPs-pZNF580 to the PCL-PIBMD constructs could provide adequate advantage in the tissue engineered vascular meshes.

## 4. Conclusions

In this study, artificial vascular meshes with rapid endothelialization performance were prepared by electrospinning and electrospraying techniques. The pEGFP-ZNF580 plasmids were successfully incorporated into PCL-PIBMD meshes by MPs aiming at rapid endothelialization. The introduction of MPs-pZNF580 could be regarded as multifunctional modification, considering the improvement of surface hydrophilicity, the increased proliferation of ECs, the reduced platelet adhesion and the relatively weak inflammatory response. The PCL-PIBMD/MPs-pZNF580 meshes exhibited good mechanical properties with tensile strength of 27 ± 3 MPa, elongation at break of 117% ± 8% and good cytocompatibility and tissue compatibility in *in vitro* and *in vivo* assays. It is reasonable to draw the conclusion that PCL-PIBMD meshes loaded with pEGFP-ZNF580 plasmids via MPs can act as potential vascular meshes in cardiovascular tissue engineering.
